# Mixed Matrix Membranes Loaded with a Porous Organic Polymer Having Bipyridine Moieties

**DOI:** 10.3390/membranes12060547

**Published:** 2022-05-25

**Authors:** Sandra Rico-Martínez, Cristina Álvarez, Antonio Hernández, Jesús A. Miguel, Ángel E. Lozano

**Affiliations:** 1 IU CINQUIMA, University of Valladolid, Paseo Belén 5, E-47011 Valladolid, Spain; sandra.rico@uva.es (S.R.-M.); lozano@ictp.csic.es (Á.E.L.); 2 Institute for Polymer Science and Technology (ICTP-CSIC), Juan de la Cierva 3, E-28006 Madrid, Spain; 3 Surfaces and Porous Materials (SMAP, UA-UVA_CSIC), Associated Research Unit to CSIC, University of Valladolid, Paseo Belén 7, E-47011 Valladolid, Spain; antonio.hernandez@uva.es

**Keywords:** gas separation, polyimides, mixed matrix membranes, porous organic polymers, CO_2_ capture

## Abstract

Mixed matrix membranes (MMMs), derived from three aromatic polyimides (PIs), and an affordable porous organic polymer (POP) having basic bipyridine moieties were prepared. Matrimid and two fluorinated polyimides, which were derived from 4,4′-(hexafluoroisopropylidene)diphthalic anhydride and 2,2′-bis(4-aminophenyl)hexafluoropropane (6F6F) or 2,4,6-trimethyl-m-phenylenediamine (6FTMPD), were employed as polymer matrixes. The used POP was a highly microporous material (surface area of 805 m^2^ g^−1^) with excellent thermal and chemical stability. The MMMs showed good compatibility between the PIs and POP, high thermal stabilities and glass transition temperatures superior to those of the neat PI membranes, and good mechanical properties. The addition of POP to the matrix led to an increase in the gas diffusivity and, thus, in permeability, which was associated with an increase in the fractional free volume of MMMs. The increase in permeability was higher for the less permeable matrix. For example, at 30 wt.% of POP, the permeability to CO_2_ and CH_4_ of the MMMs increased by 4- and 7-fold for Matrimid and 3- and 4-fold for 6FTMPD. The highest CH_4_ permeability led to a decrease in CO_2_/CH_4_ selectivity. The CO_2_/N_2_ separation performance was interesting, as the selectivity remained practically constant. Finally, the POP showed no molecular sieving effect towards the C_2_H_4_/C_2_H_6_ and C_3_H_6_/C_3_H_8_ gas pairs, but the permeability increased by about 4-fold and the selectivity was close to that of the matrix. In addition, because the POP can form metal ion bipyridine complexes, modified POP-based MMMs could be employed for olefin/paraffin separations.

## 1. Introduction

The urgent need to reduce CO_2_ emissions has led to an intense search for new materials to improve the capture of CO_2_ emissions from fossil energy use [1,2,3]. At the same time, it is necessary to advance in other separations, such as the removal of CO_2_ from natural gas because it is a crucial step before making it available to the market [4,5,6].

In this regard, membrane technology is receiving increasing attention as it has shown great potential in industrial separations [7,8]. Polymer membranes are by far the most widely studied materials for gas separation processes, as they have good mechanical stability, are easy to process, and can be formed from a large number of polymer materials. However, they usually present a permeability/selectivity trade-off and suffer from plasticization, physical aging, and competitive adsorption phenomena [7,9]. Nowadays, a great effort is being made in the search for polymer membranes in which these drawbacks are minimized or suppressed [10,11].

In this context, a thoughtful design of mixed matrix membranes (MMMs) should combine, synergistically, the easy processability, good mechanical stability, and low cost of polymer matrices and the high performance in terms of permeability and/or selectivity of porous fillers to overcome the permeability/selectivity trade-off of neat polymer membranes [11,12,13]. However, effects such as poor matrix/filler adhesion, partial pore blockage, and polymer rigidification lead to the formation of materials having poor properties. Thus, the search for porous materials that allow for obtaining high-performance gas separation membranes is currently of great interest [11,14,15,16]. Among them, metal organic frameworks (MOFs) [17,18,19,20], covalent organic frameworks (COFs) [21], hyper-crosslinked polymers (HCPs) [22], and porous organic polymers (POPs) [23,24,25,26,27,28,29,30] are being widely studied as new fillers in MMMs.

Recently, our group has developed a low-cost synthetic methodology to prepare POPs by electrophilic aromatic substitution reaction between a ketone having electron-withdrawing groups and polyfunctional and rigid aromatic compounds [31]. The obtained POPs were highly microporous materials, with Brunauer-Emmett-Teller (BET) surface areas up to 800 m^2^g^−1^ and CO_2_ uptakes superior to 207 mg g^−1^ at 273 K and 1 bar. In addition, they presented outstanding thermal stability (superior to 450 °C) and exceptional chemical resistance. Among these POPs, the porous materials derived from triptycene and isatin (TRP-Is) or trifluoroacetophenone (TRP-TFAP) have been employed to prepare MMMs using very different polymer matrixes: linear polyimides [32] and thermally rearranged (TR) polybenzoxazoles [30,33]. All of the resulting MMMs showed high thermal stability, good mechanical properties, and enhanced CO_2_/CH_4_ and CO_2_/N_2_ separation performances.

In this work, we employed an analogous POP as filler, which is derived from 4,5-diazafluoren-9-one (DAFO) and 1,3,5-triphenylbenzene (135TPB) to prepare MMMs. This POP (135TPB-DAFO) contains bipyridine functionality, which should exhibit good affinity for acid gases, such as CO_2_; thus, it would be expected that the MMMs derived from 135TPB-DAFO would show an enhanced separation performance for gas mixtures where the CO_2_ gas was one of the components. Moreover, 135TPB-DAFO has the advantage of being a material more affordable compared to derivatives of other rigid and aromatic entities, such as triptycene.

Three aromatic polyimides with different fractional free volume (FFV), which will cover a wide range of gas separation performance, were chosen to prepare 135TPB-DAFO-based MMMs. Thus, they were prepared from a commercial polyimide with low FFV, such as Matrimid [32,34,35], and two fluorinated polyimides prepared for this work, which were derived from 4,4′-(hexafluoroisopropylidene)diphthalic anhydride (6FDA) and 2,2′-bis(4-aminophenyl)hexafluoropropane (6FpDA), 6F6F polyimide, with medium FFV [32,36], or 2,4,6-trimethyl-m-phenylenediamine (TMPD), 6FTMPD polyimide, with high FFV [32,37]. MMMs were prepared with loadings of 20 and 30 wt.% of total solid (loading + polymer), and even 40 wt.% for the membranes derived from Matrimid, using a casting technique. The MMMs were characterized by common techniques such as scanning electron microscopy and X ray diffraction, and their thermal and mechanical properties were studied. The application of these MMMs for CO_2_ separation over CH_4_ and N_2_ was evaluated.

Finally, we carried out a preliminary study to know the potential application of these materials in olefin/paraffin separation. The straightforward modification of 135TPB-DAFO to form metal ion bipyridine complexes could be used to prepare transport-facilitated MMMs that could exhibit a good capacity to separate these gas mixtures of high industrial interest.

## 2. Materials and Methods

### 2.1. Materials

4,5-Diazofluoren-9-one (DAFO) was synthesized in our laboratory following the synthetic route described in the literature [38] and modified by us [39]. The detailed synthesis of DAFO is described in the S1 Section in the Supporting Information. The 4,4′-(hexafluoroisopropylidene)diphthalic anhydride (6FDA, 99% of purity) was supplied by TCI Europe (Zwijndrecht, Belgium), and it was purified by sublimation at 220 °C under high vacuum prior to use. The 2,2′-bis(4-aminophenyl)hexafluoropropane (6FpDA, 99% of purity) and 2,4,6-trimethyl-m-phenylenediamine (TMPD, 96% of purity) were purchased from Cymit Química (Barcelona, Spain) and Sigma-Aldrich (St. Louis, MO, USA), respectively, and purified by sublimation at 220 °C and 110 °C before use. Matrimid 5218 was kindly gifted by Huntsman. Chlorotrimethyl silane (CTMS, >98% of purity) and N,N-dimethylaminopyridine (DMAP, ≥99% of purity) were purchased from TCI. Anhydrous dimethylacetamide (DMAc, 99.8% of purity), anhydrous pyridine (Py, 99.5% of purity), acetic anhydride (97% of purity), and 1,3,5-triphenylbenzene (135TPB, >99% of purity) were purchased from Alpha Aesar (Haverhill, MA, USA). The trifluoromethanesulfonic acid (TFSA, 99% of purity) was purchased from Fluorochem (Glossop, UK).

### 2.2. Methods

^1^H nuclear magnetic resonance (NMR) spectra were recorded on a Bruker Advance 500 apparatus (Billerica, MA, USA) working at 500 MHz, using deuterated chloroform (CDCl_3_) as the solvent. Solid state ^13^C cross-polarization magic angle spinning NMR spectra (CP-MAS ^13^C NMR) were recorded on a Bruker Avance 400 spectrometer equipped with an 89-mm wide bore and a 9.4-T superconducting magnet. The spectrometer operated at a Larmor frequency of 100 MHz using a contact time of 1 ms and a delay time of 3 s. The sample was spun at 9 kHz. Attenuated Total Reflectance-Fourier Transform Infrared (ATR-FTIR) spectra were registered on a PerkinElmer Spectrum RX-I FTIR spectrometer (Waltham, MA, USA). Differential scanning calorimetric (DSC) measurements were carried out on a TA Instruments DSC Q-2000 Analyzer (New Castle, DE, USA). Experiments were conducted at a heating rate of 20 °C min^−1^ under nitrogen atmosphere (50 mL min^−1^), using 6–8 mg of sample placed in aluminum pans. The glass transition temperature (Tg) was taken as the middle point of the endothermic step on the second heating scan. Thermogravimetric analyses (TGA) were performed on a TA Q-500 thermobalance under nitrogen atmosphere (60 mL min^−1^). High-resolution dynamic thermogravimetric analyses (Hi-Res^TM^ TGA) were carried out at 20 °C min^−1^ from 30 to 850 °C, with sensitivity and resolution parameters of 1 and 4, respectively. Wide-angle-X-ray scattering (WAXS) patterns were recorded in the reflection mode at room temperature, using a Bruker D8 Advance diffractometer provided with a Goebel Mirror and a PSD Vantec detector. CuK_α_ (wavelength λ = 1.54 Å) radiation was used. A step-scanning mode was employed for the detector, with a 2θ step of 0.024° and 0.5 s per step. Inherent viscosities of polyimides were measured at 30 °C with an Ubbelohde viscometer, using NMP as the solvent at 0.5 g dL^−1^ concentration. Polymer solubility tests were carried out by dissolving 10 mg of sample in 1 mL of solvent at room temperature. Scanning electron microscopy (SEM) images were taken with a QUANTA 200 FEG ESEM (FEI, Hillsboro, OR, USA) on Au-metallized samples operating at an acceleration voltage of 1.5 kV in high vacuum and using the detection of secondary electrons method. The density of the membranes (ρ) was determined from Archimedes’ principle, using a top-loading electronic XS105 dual range Mettler Toledo balance provided with a density measurement kit (Columbus, OH, USA). The samples were sequentially weighed in air and into high purity isooctane at 25 °C. Six density measurements were made for each sample. The density was calculated from Equation (1):

(1) $$\rho =\rho_{\mathrm{liquid}}\frac{w_{\mathrm{air}}}{w_{\mathrm{air}}-w_{\mathrm{liquid}}}$$

where $\rho_{\mathrm{liquid}}$ is the density of isooctane, $w_{\mathrm{air}}$ is the weight of the sample in air, and $w_{\mathrm{liquid}}$ is its weight when submerged in isooctane. From the density data, the fractional free volume (FFV) was estimated using Equation (2):

(2) $$\mathrm{FFV}=\frac{V-1.3\left[\varphi_{\mathrm{POP}}V_{W}^{\mathrm{POP}}+\left(1-\varphi_{\mathrm{POP}}\right)V_{W}^{\mathrm{PI}}\right]}{V}$$

where V (=1/ρ) is the specific volume of the membrane, $V_{W}^{\mathrm{PI}}$ and $V_{W}^{\mathrm{POP}}$ are the van der Waals volumes of neat polyimide and neat POP, respectively, which were calculated by molecular modeling of repeat units applying the semiempirical Austin Model (AM1) in the Hyperchem Molecular Modeling Program [40], and $\varphi_{\mathrm{POP}}$ is the volume fraction of POP, calculated according to Equation (3):

(3) $$\varphi_{\mathrm{POP}}=\frac{w_{\mathrm{POP}}}{w_{\mathrm{POP}}+\left(\frac{\rho^{\mathrm{POP}}}{\rho^{\mathrm{PI}}}\right)\left(1-w_{\mathrm{POP}}\right)}$$

where $w_{\mathrm{POP}}$ is the weight fraction of POP in the membrane, $\rho^{\mathrm{PI}}$ is the density of the neat polyimide membrane, and $\rho^{\mathrm{POP}}$ is the density of the POP (1.533 g cm^−3^), which was estimated from its skeletal density (1.113 g cm^−3^), measured by helium pycnometry (Accupyc 1330 device, Micromeritics Instrument Corporation, Norcross, GA, USA), and its total pore volume (0.42 cm^3^ g^−1^), obtained from low-pressure N_2_ adsorption isotherms at −196 °C. The mechanical properties of the membranes were evaluated under uniaxial tensile tests at room temperature using an MTS Synergie-200 testing machine (Eden Prairie, MN, USA) equipped with a 100 N load cell. Rectangular pieces of 5 mm width and 25 mm length were subjected to a tensile load applied at 5 mm min^−1^ until fracture. Sorption tests for pure CO_2_ were conducted for solid porous materials using a Cahn D2000 microbalance at 25 °C. Approximately 80 mg of sample were placed on the sample pan, and the whole system was evacuated (10^−2^ mbar) for 24 h. Next, CO_2_ at a specific pressure (0.930 bar) was fed into the system, and the sample started to sorb the gas until the equilibrium was achieved. From the weight gain, the amount of CO_2_ adsorbed in the sample was calculated after accounting for buoyancy correction. Pure gases He, O_2_, N_2_, CH_4_, and CO_2_ permeability was measured at 30 °C and an upstream pressure of 3 bar, using a constant volume/variable pressure apparatus. Before starting the permeation experiment, the membrane inside the permeation cell was kept under high vacuum overnight. The increase in the downstream pressure was recorded as a function of time. All gases were allowed to permeate until steady state (SS) conditions were reached. The permeability coefficient (P) was determined from the slope of downstream pressure vs. time at steady state using the Equation (4):

(4) $$P=\frac{273\mathrm{Vl}}{76{\mathrm{ATp}}_{0}}\left[{\left(\frac{\mathrm{dp}\left(t\right)}{\mathrm{dt}}\right)}_{\mathrm{ss}}-{\left(\frac{\mathrm{dp}\left(t\right)}{\mathrm{dt}}\right)}_{\mathrm{leak}}\right]$$

where V is the downstream volume (cm^3^), l and A are the thickness (cm) and the effective area of the membrane (cm^2^), T is the temperature (K), $p_{0}$ is the upstream pressure (bar), (dp(t)/dt)_ss_ is the steady state rate of the pressure-rise (mbar s^−1^), and (dp(t)/dt)_leak_ is the system leak rate (mbar s^−1^), which was less than 1% of (dp(t)/dt)_ss_. Permeability is expressed in Barrer (1 Barrer = 10^−10^ cm^3^ (STP) cm cm^−2^ s^−1^ cmHg^−1^). The diffusion coefficient (D) was determined using Equation (5):

(5) $$D=\frac{l^{2}}{6\theta }$$

where θ is the time lag (intercept of the time axis with the extrapolated linear steady state part of the curve). The solubility coefficient (S) was indirectly estimated from S = P/D, assuming the validity of the solution-diffusion mechanism through the membrane. The ideal selectivity for a gas pair (α_A/B_) was calculated from their pure gas permeability (P_A_ and P_B_) using Equation (6):

(6) $$\alpha_{A/B}=\frac{P_{A}}{P_{B}}$$

### 2.3. Synthesis of Polyimides

The aromatic polyimides, 6F6F and 6FTMPD, were prepared by a two-step polycondensation reaction of equimolecular amounts of 6FDA dianhydride and 6FpDA or TMPD diamine, by employing a base-assisted in-situ silylation method [32,41,42,43]. The general procedure was the following: in a 100 mL three-necked flask, equipped with a mechanical stirrer and under nitrogen atmosphere, 9.0 mmol of diamine (6FpDA or TMPD) was dissolved in 9.0 mL of anhydrous DMAc. Afterward, the solution was cooled to 0 °C, and the correspondent amounts of CTMS (2.1 mol/mol diamine) and anhydrous Py (2.1 mol/mol diamine) were added. Next, the temperature was allowed to raise to room temperature to ensure the diamine silylation. The solution was then cooled again to 0 °C, and 9.0 mmol of 6FDA, followed by 9.0 mL of DMAc, and then DMAP (0.21 mol/mol Py) were added. The temperature was raised to room temperature, and the reaction proceeded overnight in order to form the poly(amic acid). Subsequently, the poly(amic acid) was chemically imidized by adding acetic anhydride (8 mol/mol diamine) and Py (8 mol/mol diamine) to the solution. The reaction was stirred at room temperature for 5 h, 50 °C for 2 h, and then cooled to room temperature. Then, the polyimide was precipitated in distillated water, filtered, and consecutively washed with water and a water/ethanol (1/1) mixture. Finally, it was dried in a vacuum oven at 120 °C for 24 h. The inherent viscosity of the polyimides was high enough (0.66 for Matrimid, 0.70 for 6F6F and 0.56 dL g^−1^ for 6FTMPD) to be processed as films of MMMs having good mechanical properties.

### 2.4. Synthesis of Porous Organic Polymer

The porous organic polymer, 135TPB-DAFO, was obtained in quantitative yield by reacting stoichiometric amounts of 135TPB and DAFO in a superacidic media, TFSA, following the methodology reported previously [31,39]. Details on the synthesis and a complete characterization of 135TPB-DAFO are provided in the Sections S2 and S3 in the Supporting Information.

### 2.5. Preparation of Mixed Matrix Membranes

Mixed matrix membranes with different POP loads (20, 30, and 40 wt.%) were prepared following the procedure reported previously [32]. As an example, the preparation of MMMs containing 20 wt.% of 135TPB-DAFO is described as follows: a suspension of the POP (100 mg) in tetrahydrofuran (THF, 7.0 mL) was dispersed by stirring for 24 h at room temperature, followed by sonication for 20 min with a 130 W ultrasonic probe (Vibra Cell^TM^ 75186, Sonics and materials INC, Newtown, CT, USA) operating at 20% maximum amplitude. The procedure consisted of 40 cycles of 20 s ultrasonic exposures and a 10 s cool-down so that the particles could be entirely dispersed. Then, 1 mL of a previously prepared polymer solution (400 mg of polymer in 3.0 mL of THF) was added to the stirring suspension of POP, which was sonicated in the same previous conditions for a further 10 min (20 cycles) before adding the rest of the polymer solution. After stirring the suspension for a further 15 min, it was poured into a glass ring placed on a leveled glass plate, covered with a watch-glass, and left at room temperature overnight to remove most of the solvent. Finally, the films were peeled off from the glass plate and subjected to the following thermal treatment under vacuum conditions: 60 °C for 1 h, 100 °C for 2 h, 120 °C for 2 h, 150 °C for 2 h, and 180 °C for 12 h, and they were then allowed to cool slowly. After this thermal treatment, they were then gradually heated up to 315 °C and allowed to cool slowly to remove the entrapped solvent. Finally, TGA of the membranes was performed to check the total removal of the solvent.

For comparative purposes, neat polyimide membranes were prepared by dissolving 400 mg of polymer in 10 mL of THF. The polymer solution was filtered through a 3.1-μm fiberglass filter (Symta 30MM Syr Filter GMF 3.1 UM, Symta SAL, Madrid, Spain) and poured into a glass ring placed on a leveled glass plate. The membranes were left overnight at room temperature, peeled off from the glass plate, and then subjected to the same thermal treatment as described above.

The neat polyimide membranes and MMMs will be hereinafter referred to as PI and PI-x, where PI will be Matrimid, 6F6F, or 6FTMPD, and x the percentage by weight of 135TPB-DAFO. Films with a thickness ranging from 48 to 70 µm were obtained (Table S1 in the Supporting Information).

## 3. Results and Discussion

### 3.1. Characterization of MMMs

**Figure 1:**
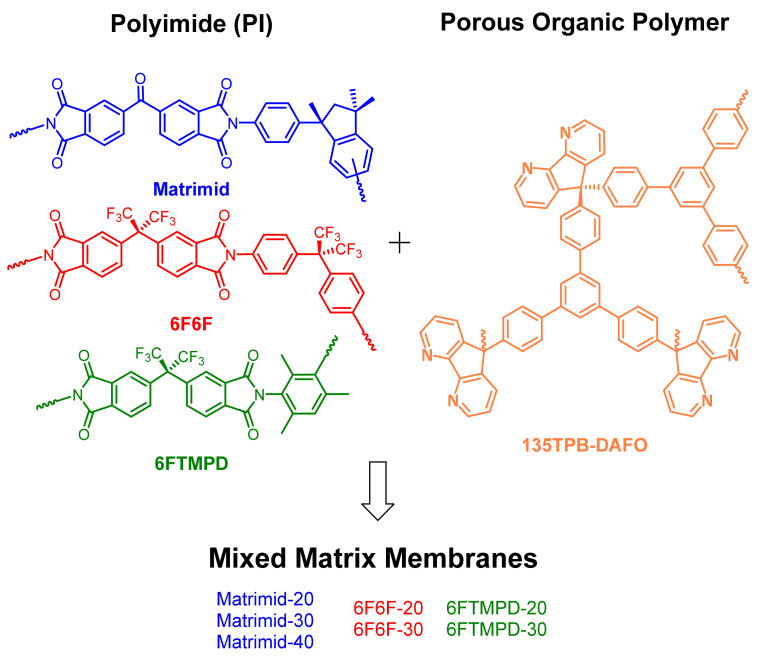
Chemical structures of polyimides and POP used to prepare the MMMs.

Three families of MMMs were prepared using three very different polyimides in their gas separation performance—Matrimid (low permeability and high selectivity), 6F6F (moderate permeability and selectivity), and 6FTMPD (high permeability and low selectivity)—as matrices and different loadings of 135TPB-DAFO. The loading percentage in the MMMs was superior to 15 wt.% of the total weight of the membrane, according to the results reported for other analogue materials [30,32,33]. The chemical structures of polyimides and POP and acronyms used to refer to the neat polyimide membranes and MMMs are shown in Figure 1.

**Figure 2:**
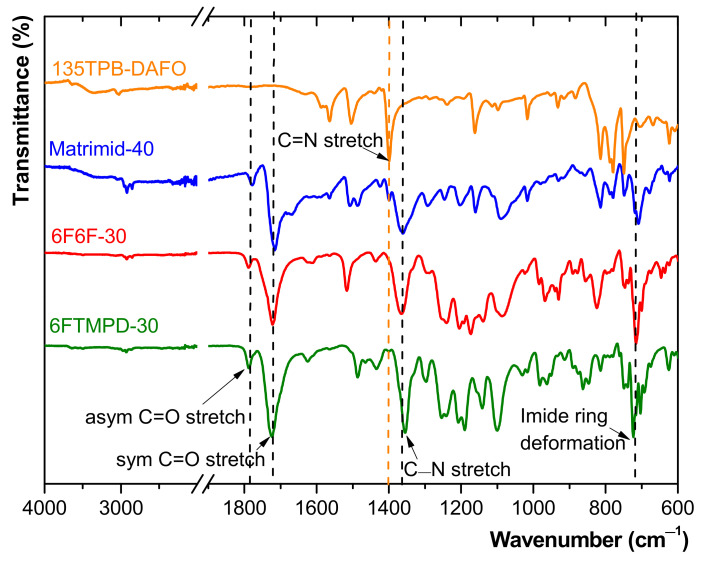
ATR-FTIR spectra of some of the prepared MMMs.

The ATR-FTIR technique was used to characterize the neat polyimide membranes and MMMs. All of the spectra, grouped according to the polyimide matrix, are shown in Figures S6–S8 in the Supporting Information. As an example, Figure 2 shows the ATR-FTIR spectra of the 135TPB-DAFO and the MMMs containing the highest POP content: Matrimid 40, 6F6F-30, and 6FTMPD-30. All the spectra of the MMMs showed the characteristic absorption bands of imide groups: 1780 (asymmetric C=O stretch), 1720 (symmetric C=O stretch), 1360 (C−N stretch), and 725 cm^−1^ (imide ring deformation). However, the absorption bands of POP were not observed in the MMMs. Only a small absorption band at 1560 cm^−1^, which can be assigned to the C=N stretching vibration from DAFO [39], was seen in the Matrimid-40 spectra.

**Figure 3:**
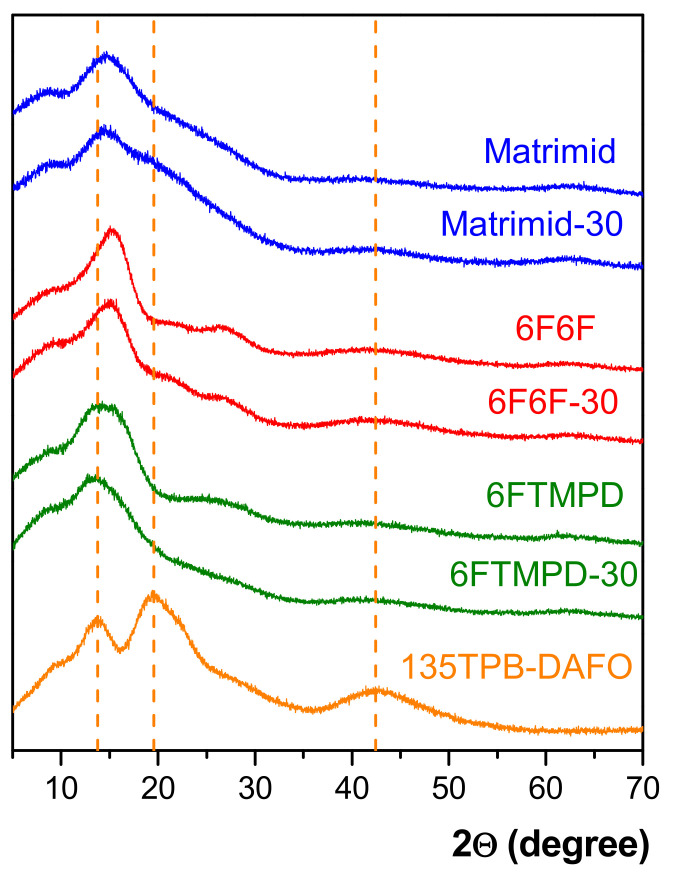
WAXS patterns of POP, neat polyimide membranes, and MMMs containing 30 wt.% of the 135TPB-DAFO loading.

The amorphous nature of POP, neat polyimide membranes, and MMMs was confirmed by WAXS. As an example, the patterns of the MMMs at 30 wt.% of the 135TPB-DAFO loading are compared with those of the neat polyimide membranes and POP in Figure 3. The 135TPB-DAFO showed an amorphous halo with three well-defined maxima around 13.8, 19.6, and 42.4°, indicating some regularity in the chain’s packing, presumably due to the flat and symmetrical triangular shape of the 135TPB moiety. Assuming that the position of these peaks can be related to the packing density of the membrane, and by applying Braggs’s law (λ = 2dsinΘ, with Θ being the scattering angle), these maxima were associated with the most probable intersegmental distances (d) of 0.64, 0.45, and 0.21 nm. In the case of the neat polyimide membranes, only a well-defined maximum was observed at 14.4 for Matrimid, 15.5 for 6F6F, and 14.7° for 6FTMPD; these scattering angles corresponded to the intersegmental distances of 0.61, 0.57, and 0.60 nm, respectively. When the WAXS patterns of the MMMs were compared with those of the pristine membrane, it was observed that the shape of the amorphous halo hardly changed. Thus, the incorporation of 135TPB-DAFO in the polyimide matrix did not seem to change the polymer chains’ packing of MMMs relative to the pristine membranes from our WAXS data.

The FFV was estimated from the density measurements of the membranes using Equation (2). The FFV values of neat polyimide membranes and MMMs are given in Table 1. It was observed that the FFV of Matrimid-30 and Matrimid-40 increased by 1.17-fold relative to its pristine membrane, while that for 6F6F-based MMMs hardly increased and for 6FTMPD-based MMMs decreased by 0.93-fold. These findings suggest that the addition of 135TPB-DAFO to the matrix with the lowest FFV (i.e., Matrimid) led to a higher increase in FFV of their MMMs.

**Figure 4:**
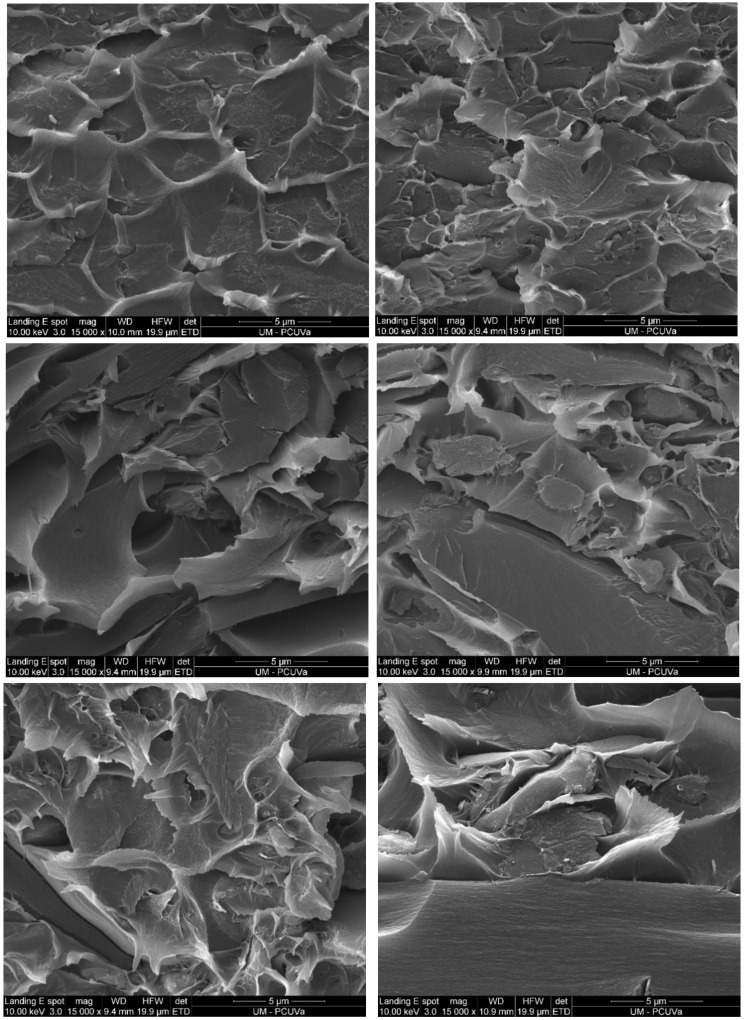
SEM micrographs of MMMs containing 20 (**left**) and 30 wt.% (**right**) of the 135TPB-DAFO loading from Matrimid (**up**), 6F6F (**middle**), and 6FTMPD (**bottom**).

SEM images made from the cross-section of the MMMs and the neat polyimide membranes are shown in Figure 4 and Figure S9 in the Supporting Information. The compatibility of the 135TPB-DAFO particles with all the polyimide matrixes seemed to be good; even the MMMs with the highest POP content seemed to retain the good compatibility between the POP and polyimide matrix (SEM image of Matrimid-40 is shown in Figure S10 in the Supporting Information). The visible micrometer-sized cavities that appeared in the SEM images could be caused by the ductile fracture mode of the membranes during the cryo-fracturing.

### 3.2. Thermal Properties of MMMs

Thermal properties of neat polyimide membranes were studied by DSC and TGA techniques. The onset degradation temperatures (T_d_), char yields at 800 °C (R^800^), and glass transition temperatures (T_g_) of all the membranes are listed in Table 1.

**Figure 5:**
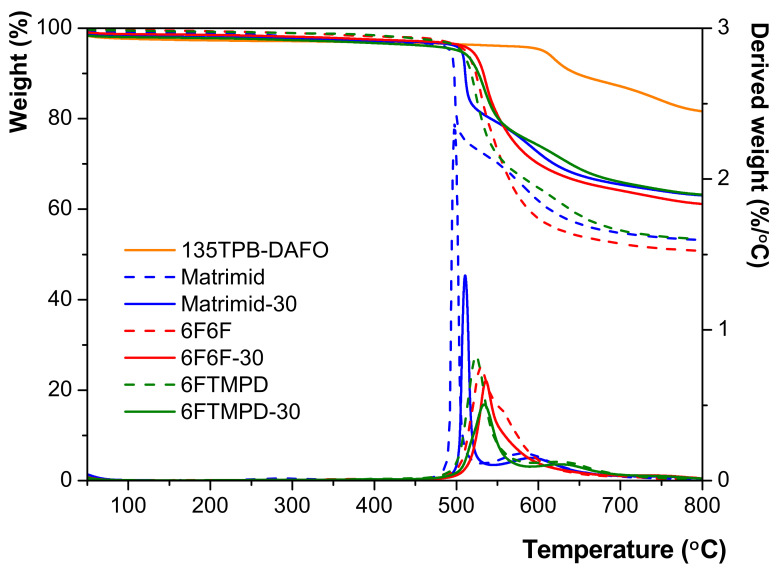
TGA curves of the 135TPB-DAFO, neat polyimide membranes, and MMM containing 30 wt.% of the 135TPB-DAFO POP.

The thermal stabilities of the MMMs were studied by TGA under atmosphere nitrogen. The thermograms of all the membranes are depicted in Figure S11 in the Supporting Information. As an example, Figure 5 shows the thermograms for the MMMs containing 30 wt.% of 135TPB-DAFO loading and the corresponding neat polyimide membranes. The thermograms of the POP and MMMs showed a loss weight above 450 °C, which was due to the generalized degradation of the materials. At lower temperatures, a weight loss was observed by about 5% related to the loss of adsorbed moisture (below 100 °C) and the remaining solvent trapped in the pores. The thermal stability of the 135TPB-DAFO (onset degradation temperature, T_d_, of 605 °C) was outstanding compared with those of other porous materials such as ZIF-8 (300 °C) [44], which is a zeolitic imidazolate framework widely employed as filler in MMMs, multifunctional porous aromatic frameworks (PAFs, T_d_ between 250 and 520 °C) [45], and porous organic polymers derived from triptycene or 135TPB with isatin or trifluoroacetophenone (T_d_ between 490 and 535 °C) [31]. In conclusion, the T_d_ of 135TPB-DAFO was around 100 °C higher than that of the pristine membranes. Despite the high thermal stability of 135TPB-DAFO, the addition of porous material to the polyimide matrix led to an increase in T_d_ of 10 °C in all the cases. The char yields were high, as expected for highly aromatic materials.

**Figure 6:**
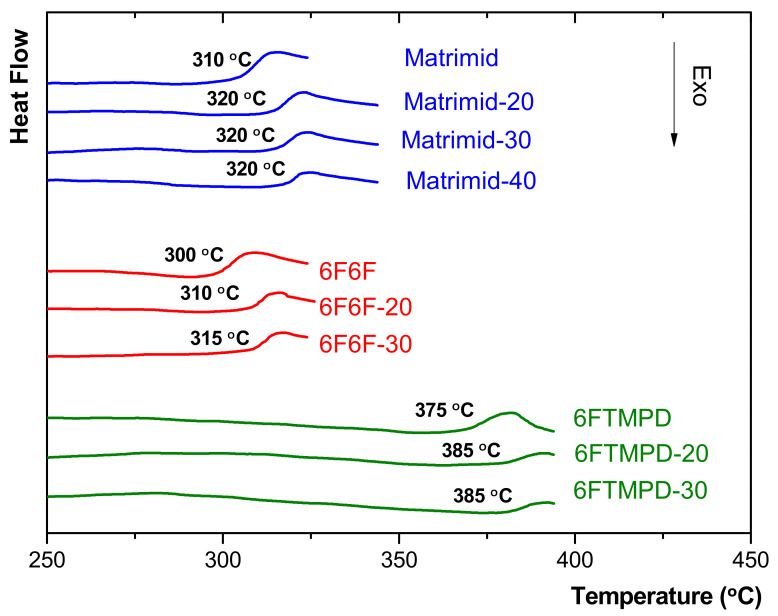
DSC curves of the neat polyimide membranes and MMMs.

Figure 6 compares the DSC curves of the MMMs relative to their neat polyimide membrane. All of the membranes showed an endotherm step associated with the glass transition, consistent with the amorphous nature of these materials (see Figure 3). The addition of the 135TPB-DAFO to the polyimide matrix increased the T_g_ relative to that of the neat polyimide membrane. For example, the T_g_ value for the MMMs containing the highest 135TPB-DAFO content increased 10 °C for Matrimid-40, 15 °C for 6F6F-30, and 5 °C for 6FTMPD-30. Moreover, the increase in T_g_ did not seem to depend on the loading amount. That is, the addition of 20, 30, or 40 wt.% of the 135TPB-DAFO loading led to a similar increase in T_g_. On the other hand, it is well-known that the restriction molecular mobility of the polymer chains causes an increase in T_g_ [29,46]. Thus, the increase in T_g_ of the MMMs relative to pristine membranes indicated a favorable interaction between the POP and the polyimides matrices.

### 3.3. Mechanical Properties of MMMs

The mechanical properties of neat polyimide membranes and MMMs derived from Matrimid, 6F6F, and 6FTMPD were obtained by tensile testing, and they are summarized in Table 2. In general, the tensile strength and elongation at the break decreased with the addition of 135TPB-DAFO to the polyimide’s matrix due to the embrittlement of MMMs [32,46,47]. In addition, the Young’s modulus decreased by about 0.6-fold for Matrimid-30 and 6F6F-30, relative to the pristine membranes, while the value for 6FTMPD-30 was similar to that of the 6FTMPD. Thus, despite that the increase in the T_g_ of MMMs compared to the pristine membranes indicated a good compatibility between the 135TPB-DAFO and the polyimide matrices, the mechanical properties suggested a possible aggregation of POP particles resulting in less contact between the loading and matrix entities. Unexpectedly, the Young’s modulus for Matrimid-40 was close to the pristine membrane. This trend could not be confirmed in the other MMMs because the 6F6F and 6FTMPD polyimides could only be loaded up to 30 wt.%, above which the membranes were brittle and difficult to handle. All prepared membranes could be measured without problems as gas separation membranes.

### 3.4. Gas Separation Properties

**Figure 7:**
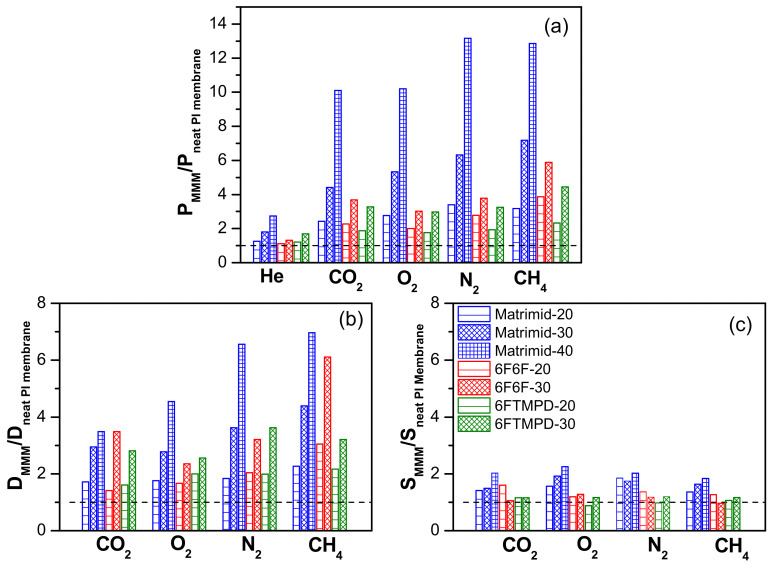
Changes in permeability (**a**), diffusivity (**b**), and solubility (**c**) for 135TPB-DAFO-based MMMs relative to their neat polyimide membrane, which have values of 1, for every tested gas.

Permeability measurements were carried out for the single gases He, O_2_, N_2_, CH_4_, and CO_2_ at 3 bar and 30 °C in the neat polyimide membranes and MMMs. The gas permeability values of the membranes and their ideal selectivity for O_2_/N_2_, CO_2_/CH_4_ and CO_2_/N_2_ are given in Table S1 in the Supporting Information. Figure 7 shows, graphically, the changes in permeability, diffusivity, and solubility coefficients of the MMMs relative to the neat polyimide membrane, to which the value of 1 was assigned, as a function of the kinetic diameters of gases in the order of He (2.6 Å) < CO_2_ (3.3 Å) < O_2_ (3.46 Å) < N_2_ (3.64 Å) < CH_4_ (3.8 Å). The gas permeability of the MMMs increased with the increasing POP content for all of the gases studied (Figure 7a). However, when comparing MMMs, it was observed that those derived from 6FTMPD (the most permeable matrix) showed the lowest increase in permeability, followed by those derived from 6F6F, and then from Matrimid (the lowest permeable matrix). In particular, the addition of POP caused a higher increase in the CH_4_ permeability—the gas with the highest molecular kinetic diameter—compared to the other gases. For example, the CH_4_ permeability increased by 7.2-fold for Matrimid-30, 5.9-fold for 6F6F-30, and 4.4-fold for 6FTMPD-30. On the other hand, the permeability of Matrimid-40 substantially increased for all of the gases, about 10-fold for O_2_ and N_2_ and 13-fold for N_2_ and CH_4_.

The increase in permeability of the MMMs was analyzed by considering the effect of the addition of POP on the diffusion (Figure 7b) and solubility (Figure 7c) coefficients. The diffusivity parameter showed the highest contribution to permeability, indicating that the increase in gas permeability was mainly due to an increase in diffusion pathways through the MMMs. For example, at 40 wt.% loading, the diffusivity contribution of Matrimid-40 to the permeability was considerably higher (5.0 for CO_2_, 4.5 for O_2_, 6.6 for N_2_, and 7.0 for CH_4_) compared to its solubility contribution (2.0 for CO_2_, 2.2 for O_2_, 2.0 for N_2_, and 1.8 for CH_4_). This behavior is consistent with that previously reported for triptycene-isatin (TRP-Is) POP-based MMMs, which showed that the POP size pore distribution should not limit diffusion gases, with diameters smaller than 0.4 nm.

**Figure 8:**
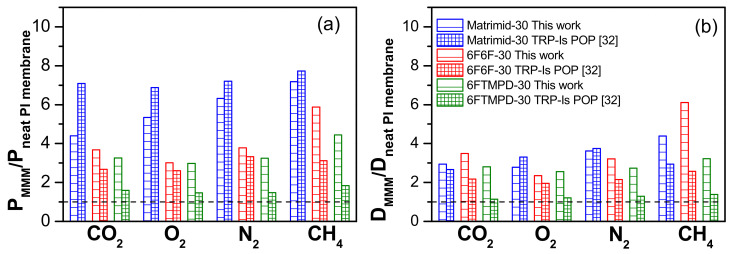
Changes in permeability (**a**) and diffusivity (**b**) for MMMs derived from POPs 135TPB-DAFO (this work) and TRP-Is [32] relative to the neat polyimide membrane, which have values of 1, for every tested gas.

For an in-depth understanding of the relationship between the compatibility of the charge with the polymer on material properties, the gas transport properties of the MMMs were compared with those of the TRP-Is POP-based MMMs [32]. Thus, Figure 8 compares the changes in permeability and diffusivity for MMMs with 30 wt.% of POP loading relative to the neat polyimide membrane. For Matrimid-based MMMs, both POPs caused the highest increase relative to the other PI-based MMMs (Figure 7a). However, the CO_2_ permeability of Matrimid-30 TRP-Is was considerably higher than that of Matrimid-30 135TPB-DAFO (7.1-fold for Matrimid-30 TRP-Is and 4.4-fold for Matrimid-30 135-DAFO), while the relative CO_2_ diffusivity was similar for both MMMs (2.9-fold for Matrimid-30 TRP-Is and 2.7-fold for Matrimid-30 135TPB-DAFO). This fact seems to indicate that the contribution of the solubility coefficient to the permeability of Matrimid-30 TRP-Is could be superior to that of Matrimid-30 135TPB-DAFO. For the other PI-based MMMs, the increase in permeability was mainly due to a higher gas diffusivity.

The main textural parameters, determined from low-pressure N_2_ adsorption isotherm at −196 °C, the skeletal density, and the maximum sorption uptake at 0.930 mbar and 25 °C (which was measured using a Cahn electrobalance) of the POPs are summarized in Table 3 (the low-pressure N_2_ adsorption/desorption isotherms are displayed in Figure S5 in Supporting Information). Both POPs showed a high N_2_ uptake at low relative pressures (P/P_0_ < 0.01), which can be related to the presence of micropores, and a low-pressure hysteresis, which is indicative of the presence of constricted micropore networks [48]. The porosity of TRP-Is was 1.7-fold higher than that of 135TPB-DAFO, showing a microporosity of 70%. This finding was consistent with the highest CO_2_ uptake of TRP-Is POP (9.0%) relative to that of 135TPB-DAFO POP (6.9%), which may support the idea of a higher solubility contribution to permeability for TRP-Is POP-based MMMs.

**Figure 9:**
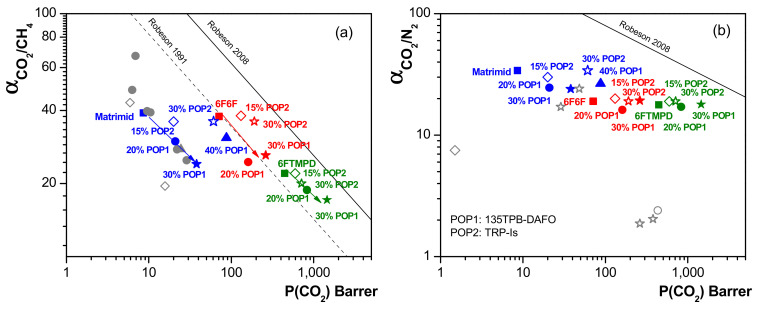
Robeson plot for CO_2_/CH_4_ (**a**) CO_2_/N_2_ (**b**) separation. Experimental data for TRP-Is POP-based MMMs were taken from [32]. Open gray symbols represent the experimental data reported for zeolite-based MMMs derived from Matrimid [49,50,51].

The gas separation performance of the MMMs studied in this work were compared to those reported for TRP-Is POP-based membranes [33], and for some Zeolite-Matrimid MMMs (with loading between 10 and 40 wt.%), which were measured in single gas permeation conditions at 25–35 °C and feed pressures varying between 5 and 10 bar [49,50,51]. The results are given on Robeson plots for several gas pairs: CO_2_/CH_4_ and CO_2_/N_2_ in Figure 9, where 1991 and 2008 upper bound lines are also included [52,53,54,55]. For the CO_2_/CH_4_ separation, it can be observed that the addition of the 135TPB-DAFO POP loading up to 30 wt.% in the polyimide matrices led to variations in MMM performance in a direction parallel to the Robeson upper limit bound. That is, the increase in CO_2_ permeability was accompanied by a decrease in CO_2_/CH_4_ selectivity (for example, 4.4- and 0.54-fold for Matrimid-40, 3.7- and 0.69-fold for 6F6F-30, and 3.26- and 0.78-fold for 6FTMPD-30). However, for the CO_2_/N_2_ separation, the selectivity did not change for the MMMs derived from the most permeable polyimides matrices (6F6F and 6FTMPD), although it decreased by 0.75-fold for Matrimid-30. These results suggest that the addition of 135TPB-DAFO to the matrix favored the diffusion of the CH_4_ gas in comparison with the addition of TRP-Is (the change in selectivity of the TRP-Is POP-based MMMs was smaller, albeit in this case, it was observed that the selectivity was constant for the Matrimid MMMs). On the other hand, at a higher loading of 40 wt.%, the CO_2_ permeability and CO_2_/CH_4_ selectivity increased. This increase in selectivity may be due to a partial blocking of the pores of 135TPB-DAFO POP, which would make the selectivity for these MMMs closer to that observed for Matrimid.

**Figure 10:**
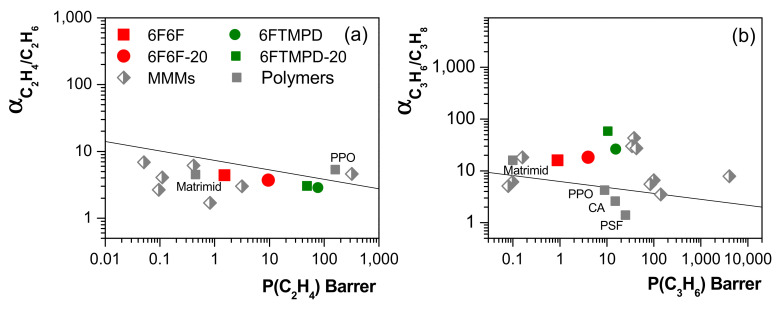
Robeson plots for C_2_H_4_/C_2_H_6_ (**a**) and C_3_H_6_/C_3_H_8_ (**b**) separations. Gray symbols represent the experimental data reported for MMMs and polymers, which were taken from [56,57,58].

From all of these results, it would be expected that the presence of 135TPB-DAFO could improve the diffusion of larger molecules through MMMs. Thus, it was considered interesting to perform a proof of concept by observing the usefulness of these materials in olefin/paraffin separations. In this context, only the MMMs derived from the most permeable polymeric matrices, 6F6F and 6FTMPD, were tested. The permeability and ideal selectivity values measured at 3 bar and 30 °C are listed in Table S2 in the Supporting Information. Figure 10 shows the Robeson plots for C_2_H_4_/C_2_H_6_ and C_3_H_6_/C_3_H_8_ separations for our MMMs, for other polymer-derived MMMs (for example: Matrimid, fluorinated polyimide, acetate cellulose (CA)) loaded with other fillers (for example: ZIF-8, SIO_2_, MOF-74 and CuBTC) [56,57], and for neat commercial polymers (e.g., Matrimid, poly(2,6-dimethyl-1,4-phenylene oxide) (PPO) and polysulfone (PSF)) [57,58]. For C_2_H_4_/C_2_H_6_ separation, it was observed that the addition of 135TPB-DAFO significantly increased the permeability relative to that of neat polyimide membranes, particularly for 6F6F-based MMMs (6.3 for 6F6F-20 and 1.6 for 6FTMPD-20) with no significant changes in selectivity. However, they did not surpass the C_2_H_4_/C_2_H_6_ upper bound, which was obtained from the experimental data of the C_2_H_4_/C_2_H_6_ separation performance of polymeric membranes to pure gases measured at 35–50 °C and 1–2 bar [57]. For C_3_H_6_/C_3_H_8_ separation, permeability and selectivity increased 4.5-fold and 1.1-fold, respectively, for 6F6F-20, while permeability increased 2.2-fold and selectivity only decreased 0.7-fold for 6FTMPD-20, relative to pure polyimide membranes. Thus, the 135TPB-DAFO appeared to show no molecular sieving effect towards the C_2_H_4_/C_2_H_6_ and C_3_H_6_/C_3_H_8_ gas pairs. Nevertheless, the 135TPB-DAFO-based MMMs exhibited enhanced permeability and improved membrane stability.

Finally, it is important to note that 135TPB-DAFO can be easily modified by the formation of metal ion bipyridine complexes, which could facilitate the olefin transport in MMMs based on π complexation [57,59].

## 4. Conclusions

A set of MMMs was prepared by combining Matrimid, 6F6F, or 6FTMPD with a high thermal and chemical stable and high microporosity POP, which bears basic bipyridine groups that can interact with CO_2_ gas. The MMMs were homogeneous with all the POP loadings studied (between 20 and 40 wt.%) and showed excellent dispersion of the particles, even at high loadings. In addition, the good compatibility between the polyimide matrix and the POP led to an increase in glass transition temperature of the MMMs relative to that of the neat polyimide membranes. The thermal stability of MMMs was close to 500 °C.

The mechanical properties of the MMMs were good enough to be tested as gas separation membranes. The gas permeability significantly increased with the increase of POP loading. The addition of POP to the matrix mainly led to a higher contribution of the gas diffusivity, which was associated with an increase in the FFV of MMMs relative to that of neat polyimide membranes. In particular, the highest increase in permeability was found for the MMMs derived from the less permeable matrix, as was the case of Matrimid.

The CO_2_/CH_4_ and CO_2_/N_2_ separation performances of MMMs were studied. For the CO_2_/CH_4_ separation, the performance of MMMs moved parallel to the 1991 upper bound due to the highest increase in CH_4_ permeability. For CO_2_/N_2_ separation, on the contrary, the performance of MMMs was closer to the 2008 upper bound.

The considerable increase in CH_4_ permeability of MMMs led us to think of the possible application of these membranes for light olefin/paraffin separations. The preliminary tests carried out in the fluorinated polyimides-based MMMs containing 20 wt.% of loading showed no molecular sieving effect towards C_2_H_4_/C_2_H_6_ and C_3_H_6_/C_3_H_8_ gas pairs. However, the addition of POP enhanced the permeability to C_2_-C_3_ hydrocarbons, while the selectivity was maintained compared with the polymer matrix. In fact, the C_3_H_6_/C_3_H_8_ separation performance of the 135TPB-DAFO-based MMMs seemed to be competitive with other reported polymer-based MMMs, in which some silicates and metal organic frameworks were employed as fillers.

From the above results, we concluded that the microporous polymer network, 135TPB-DAFO, may be successfully employed as a filler in suitable MMMs valid for CO_2_ capture in post-combustion conditions. According to these results, MMMs with promising applications for light olefin/paraffin separation could be obtained by taking advantage of the fact that the bipyridine group of 135TPB-DAFO can form metal ion complexes, which should facilitate the olefin transport.

## Figures and Tables

**Table 1:** Density, FFV, and thermal properties of neat polyimide membranes and MMMs.

Material	POP Load (wt.%)	Density (g/cm^3^)	FFV ^a^	T_d_ (°C)	R^800^ (%)	T_g_ (°C)
**135TPB-DAFO**	-	-	-	605	81	-
**Matrimid**	0	1.251 ± 0.006	0.110	495	52	310
	20	1.234 ± 0.004	0.118	500	57	320
	30	1.216 ± 0.005	0.129	505	60	320
	40	1.212 ± 0.007	0.129	505	64	320
**6F6F**	0	1.483 ± 0.014	0.208	515	50	300
	20	1.377 ± 0.006	0.212	525	58	310
	30	1.333 ± 0.007	0.211	525	60	315
**6FTMPD**	0	1.322 ± 0.007	0.218	505	53	375
	20	1.285 ± 0.005	0.208	515	59	385
	30	1.265 ± 0.004	0.204	515	62	385

^a^ Fractional free volume (FFV) was calculated using Equation (2).

**Table 2:** Mechanical properties of the MMMs and pure polyimides membranes.

Membrane	% wt. POP Load	Young’s Modulus (GPa)	Tensile Strength (MPa)	Elongation at Break (%)
**Matrimid**	0	1.6 ± 0.2	98 ± 4	11 ± 2
	20	1.8 ± 0.1	68 ± 6	5 ± 0.8
	30	0.9 ± 0.1	60 ± 11	10 ± 2
	40	1.8 ± 0.1	59 ± 9	4.4 ± 0.6
**6F6F**	0	2.1 ± 0.3	70 ± 10	5 ± 1
	20	1.6 ± 0.1	61 ± 7	4.9 ± 0.6
	30	1.4 ± 0.1	35 ± 3	3.0 ± 0.2
**6FTMPD**	0	1.4 ± 0.1	65 ± 6	5.9 ± 0.9
	20	1.4 ± 0.1	32 ± 5	2.7 ± 0.2
	30	1.3 ± 0.1	30 ± 5	2.9 ± 0.3

**Table 3:** Skeletal density and porosity parameters for 135TPB-DAFO POP.

		Low-Pressure N_2_ Adsorption Isotherm at −196 °C						
POP	ρ ^a^	S_BET_^b^	V_total_^c^	V_micro_^d^	Porosity ^e^	Microporosity ^f^	CO_2_ Uptake ^g^	Reference
**135TPB-DAFO**	1.113	806	0.42	0.24	32	57	6.9	This study
**TRP-Is**	1.234	790	0.44	0.31	55	70	9.0	[31]

^a^ Skeletal density (g cm^−3^) determined by helium pycnometry. ^b^ Specific surface area (m^2^ g^−1^). ^c^ Total pore volume (cm^3^ g^−1^) calculated at P/P_0_ = 0.99. ^d^ Micropore volume (cm^3^ g^−1^), calculated from the DR equation. ^e^ Porosity (%) defined as the V_total_ to (V_total_ + (1/density)). ^f^ Microporosity (%) defined as the V_micro_/V_total_. ^g^ CO_2_ uptake (%) determined by adsorption isotherms at 0.930 mbar and 298 K.

## Data Availability

Data is contained within the article or Supplementary Materials.

## Supplementary Materials

The following supporting information can be downloaded at: https://www.mdpi.com/article/10.3390/membranes12060547/s1, S1. Synthesis of 4,5-diazafluoren-9-one (DAFO): Scheme S1: Synthesis of DAFO; Figure S1. ^1^H-NMR of DAFO; S2. Synthesis of 135TPB-DAFO (POP): Scheme S2. Synthesis of 135TPB-DAFO; S3. Characterization of 135TPB-DAFO: Figure S2. CP/MAS ^13^C NMR spectrum of 135TPB-DAFO; Figure S3. WAXD pattern of 135TPB-DAFO; Figure S4. SEM micrograph of 135TPB-DAFO; Figure S5. Low-pressure N_2_ adsorption/desorption isotherms measurements at 77K for POPs: 135TPB-DAFO and TRP-Is; S4. Characterization of MMMs: Figure S6. ATR-FTIR spectra of POP, neat Matrimid membrane and Matrimid-based MMMs; Figure S7. ATR-FTIR spectra of POP, neat 6F6F membrane and 6F6F-based MMMs; Figure S8. ATR-FTIR spectra of POP, neat 6FTMPD membrane and 6FTMPD-based MMMs; Figure S9. SEM micrographs of neat polyimide membranes: Matrimid, 6F6F and FTMPD; Figure S10. SEM micrograph of Matrimid-40; Figure S11. TGA curves of Matrimid, 6F6F and 6FTMPD of MMMs; and S5. Gas separation properties: Table S1. Permeability, diffusivity and solubility coefficients for 135TPB-DAFO POP-based MMMs and neat polyimide membranes at 30 °C and 3 bar feed pressure. References [39,60,61,62] are cited in the Supplementary Materials.
