# Supplementary material for: Mixed Matrix Membranes Loaded with a Porous Organic Polymer Having Bipyridine Moieties
Source: Membranes (Basel). 2022 May 25;12(6):547. doi: 10.3390/membranes12060547 (PMC9228454; doi:10.3390/membranes12060547)
Supplement: Supplementary file 1 [file membranes-12-00547-s001.zip › membranes-1724302-supplementary.pdf]

membranes

Supplementary Materials

Mixed Matrix Membranes Loaded with a Porous Organic Polymer Having Bipyridine Moieties

Sandra Rico-Martínez [1], Cristina Álvarez [2,3,*], Antonio Hernández [3], Jesús A. Miguel [1,*] and Ángel E. Lozano [1,2,3]

[1] IU CINQUIMA, University of Valladolid, Paseo Belén 5, E-47011 Valladolid, Spain; sandra.rico@uva.es, jamiguel@uva.es
[2] Institute for Polymer Science and Technology (ICTP-CSIC), Juan de la Cierva 3, E-28006 Madrid, Spain; cristina.alvarez@ictp.csic.es; lozano@ictp.csic.es
[3] Surfaces and Porous Materials (SMAP, UA-UVA_CSIC), Associated Research Unit to CSIC. University of Valladolid, Paseo Belén 7, E-47011 Valladolid, Spain; antonio.hernandez@uva.es
* Correspondence: Correspondence: cristina.alvarez@ictp.csic.es; jamiguel@uva.es

Table of content

S1. Synthesis of 4,5-diazafluoren-9-one (DAFO)
S2. Synthesis of 135TPB-DAFO (POP)
S3. Characterization of 135TPB-DAFO (POP)
S4. Characterization of MMMs
S5. Gas separation properties

S1. Synthesis of 4,5-diazafluoren-9-one (DAFO)

4,5-diazafluoren-9-one (DAFO) was synthesized following the methodology described elsewhere [1], which is shown in Scheme S1. First, a mixture of H_2SO_4 (125 mL) and HNO_3 (63 mL) was carefully added to a mixture of 1,10-phenantroline (phen) (12.4 g, 68.9 mmol) and KBr (12.8 g, 108 mmol) cooled on an ice bath. The reaction was heated to reflux for 3 h and then was neutralized with an aqueous solution of NaOH. After filtering, the product was recrystallized from EtOH. 1,10-phenantroline-5,6-dione (PHADO) was obtained in 70% yield. Next, PHADO (4.00 g, 19.0 mmol) and a 0.25 M NaOH solution (200 mL) were heated to 60 °C in an open flask until the volume was halved. Afterwards, the product was filtered, washed several times with distilled water and dried under vacuum. Finally, the DAFO was purified by filtration through aluminium oxide, using chloroform as solvent, and obtained in 81% yield. ^{1}H-NMR spectrum of DAFO (500 MHz, $CDCl_3$) is shown in Figure S1; δ(ppm): 8.81 (dd, J = 5.0, 1.6 Hz, 2H), 8.00 (dd, J = 7.5, 1.6 Hz, 2H), 7.36 (dd, J = 7.5, 5.0 Hz, 2H).

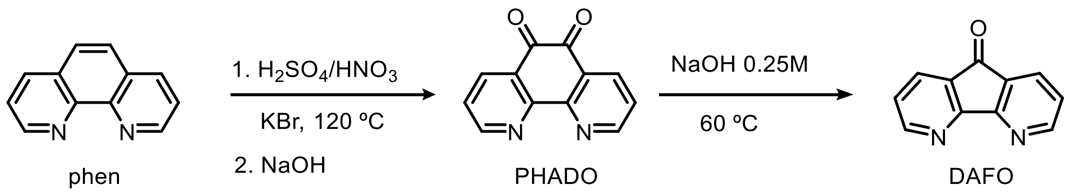

Scheme S1. Synthesis of 4,5-diazafluoren-9-one (DAFO).

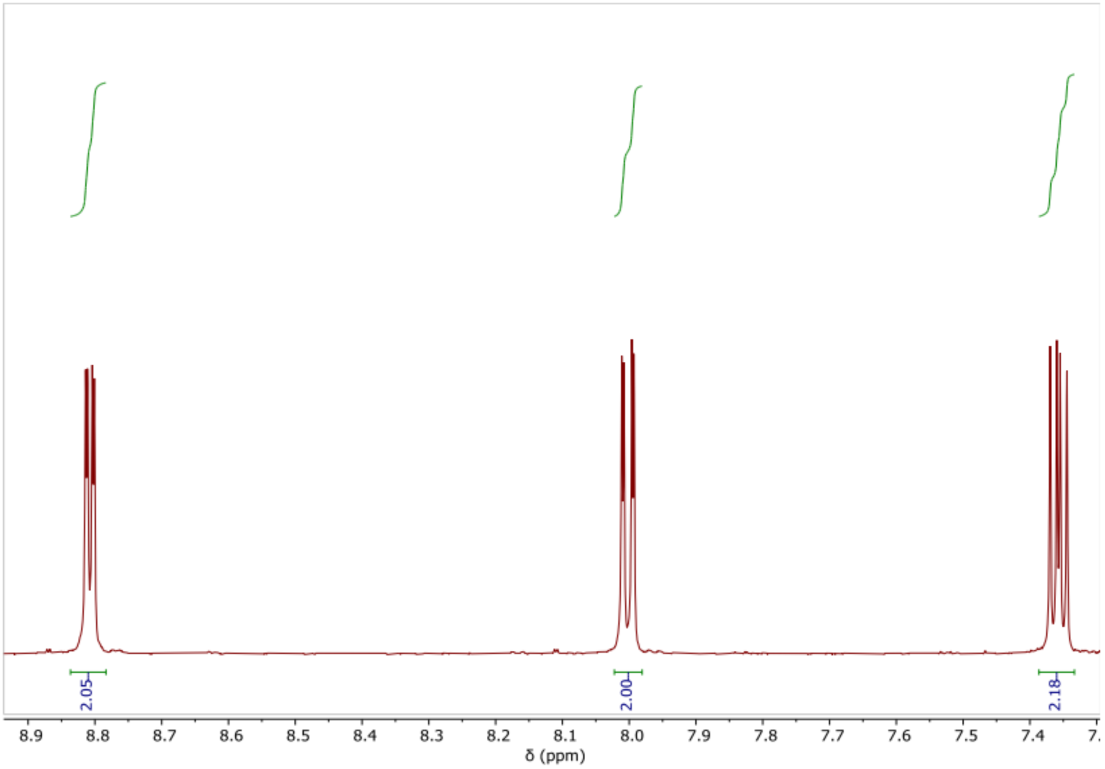

Figure S1. ^{1}H-NMR of 4,5-diazafluoren-9-one (DAFO) in $CDCl_3$.

S2. Synthesis of 135TPB-DAFO (POP)

The porous organic polymer (POP) was obtained by reacting stochiometric amounts of 1,3,5-triphenylbenzene and DAFO in TFSA following the method described as follows [1], as shown in Scheme S2.

An oven-dried three-necked Schlenk flask, equipped with a mechanical stirrer and N_2 inlet and outlet, was charged with 11 mmol of 1,3,5-triphenylbenzene (2 equivalents), and 16.5 mmol of DAFO (3 equivalents). Next, anhydrous chloroform (15 mL) was added, and the resulting mixture was stirred for 30 min at room temperature under a nitrogen blanket. Afterward, the mixture was cooled to 0 °C, and TFSA (30 mL) was dropwise added. The mixture was left to warm up to room temperature and then it was stirred for 4 days. Finally, the product was poured into water, filtered, and consecutively washed with basic water, water, methanol, acetone, and chloroform. After drying at 180 °C for 24 h under vacuum, the material was obtained as a brown powder in 99% yield.

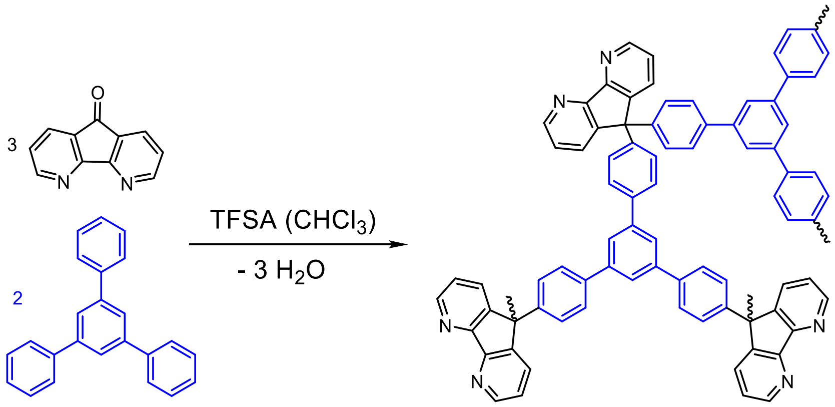

Scheme S2. Synthesis of 135TPB-DAFO.

S3. Characterization of 135TPB-DAFO (POP)

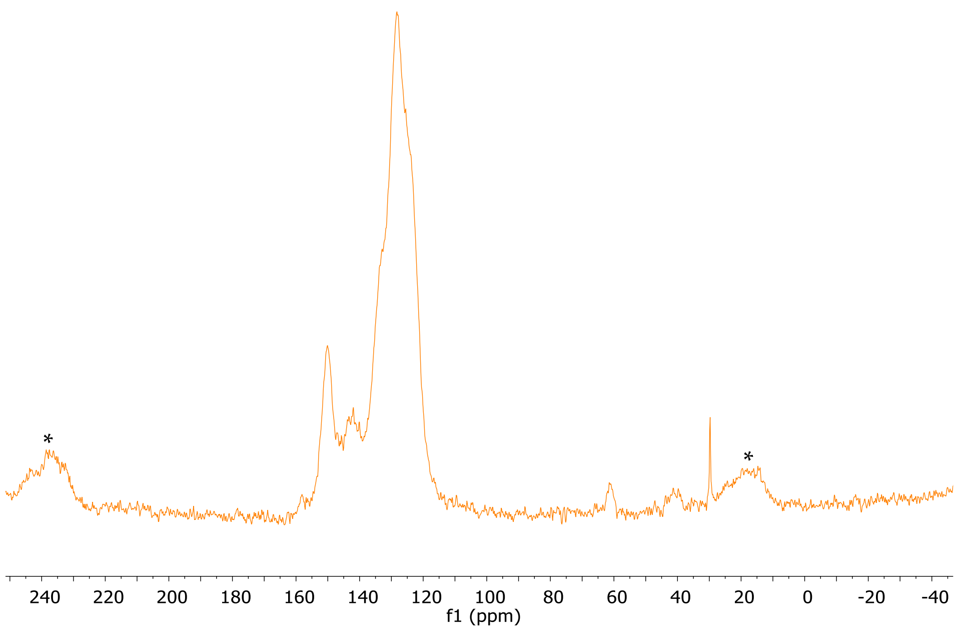

Figure S2. CP/MAS ^{13}C NMR spectrum of 135TPB-DAFO (POP). * denote spinning side bands [1].

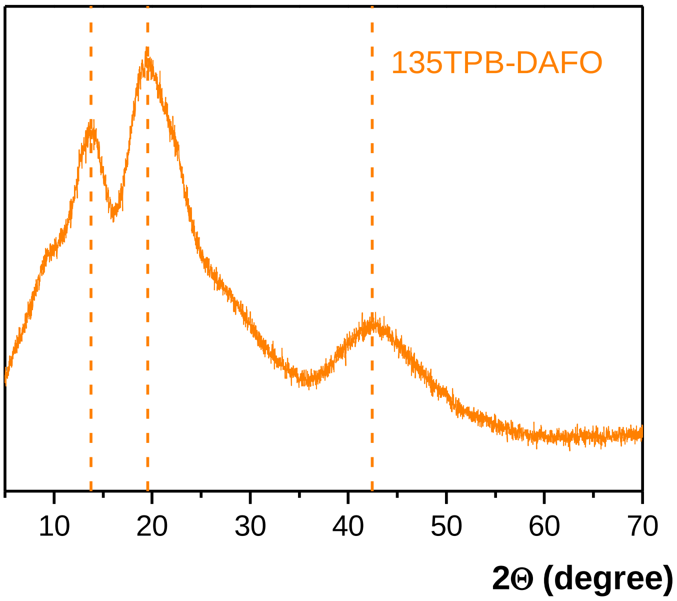

Figure S3. WAXD pattern of 135TPB-DAFO.

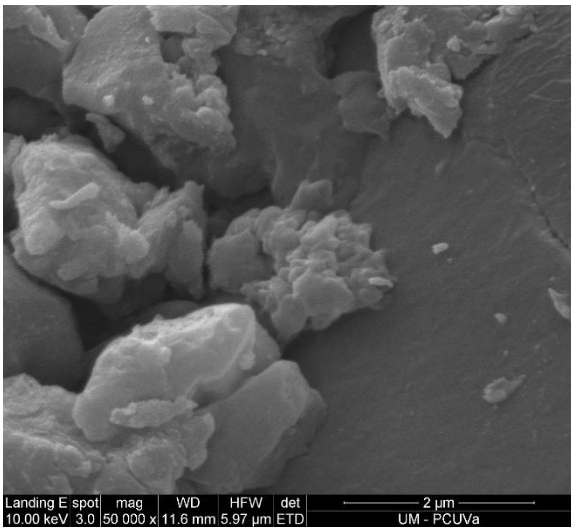

Figure S4. SEM micrograph of 135TPB-DAFO.

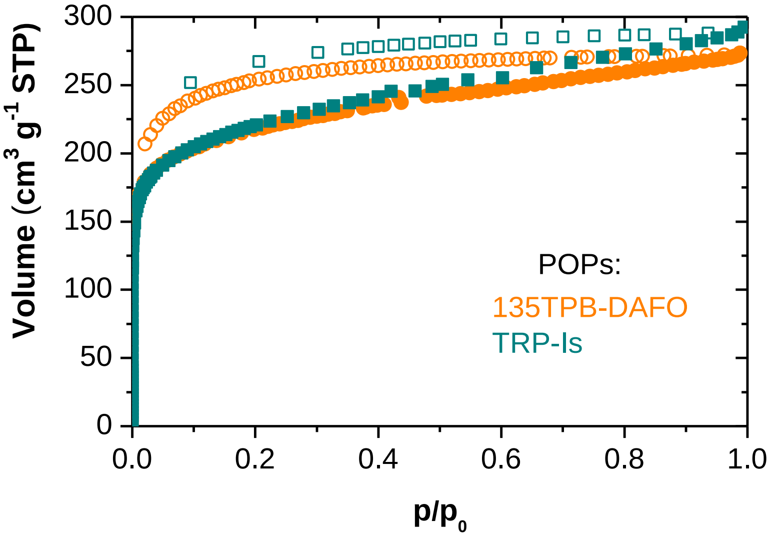

Figure S5. Low-pressure N_2 adsorption (full symbols)/desorption (empty symbols) isotherms measured at 77 K for POPs: 135TPB-DAFO (orange) and TRP-Is (green).

S4. Characterization of MMMs

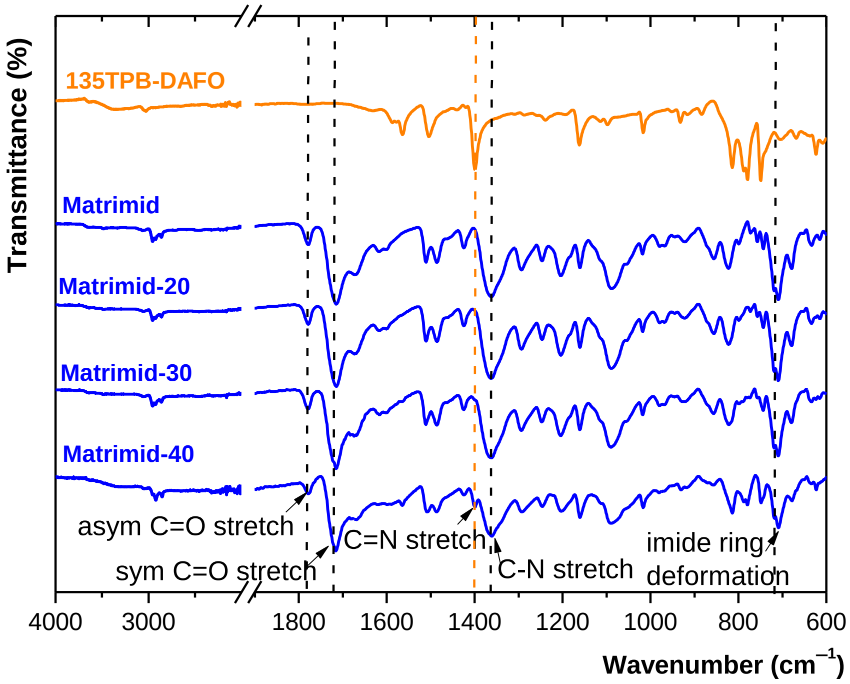

Figure S6. ATR-FTIR spectra of POP, neat Matrimid membrane and Matrimid-based MMMs.

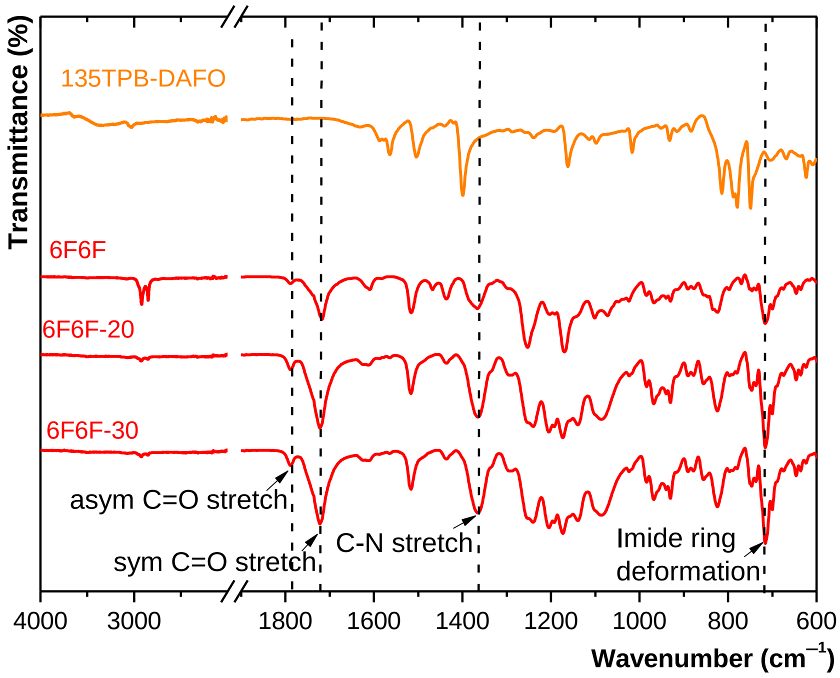

Figure S7. ATR-FTIR spectra of POP, neat 6F6F membrane and 6F6F-based MMMs.

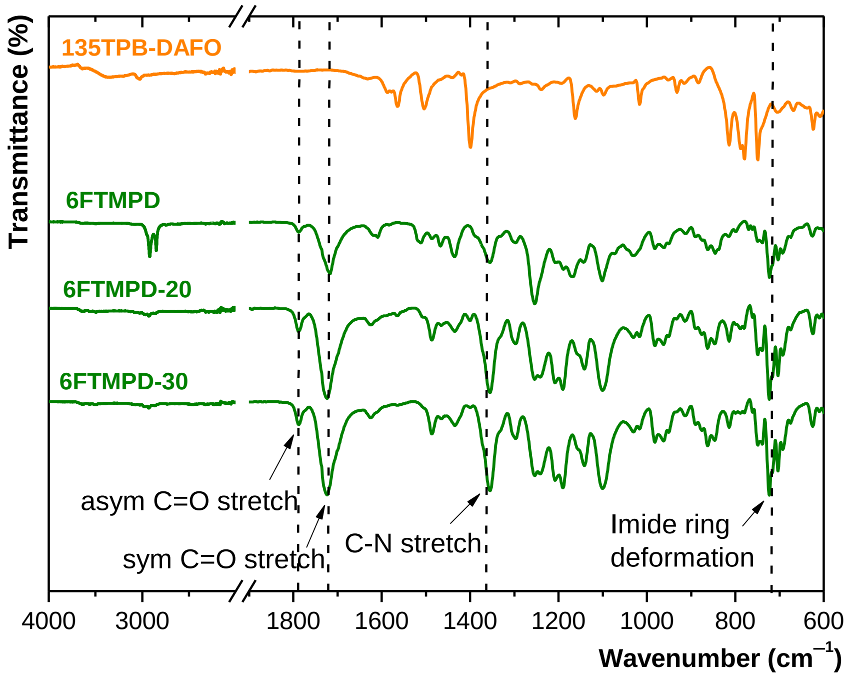

Figure S8. ATR-FTIR spectra of POP, neat 6FTMPD membrane and 6FTMPD-based MMMs.

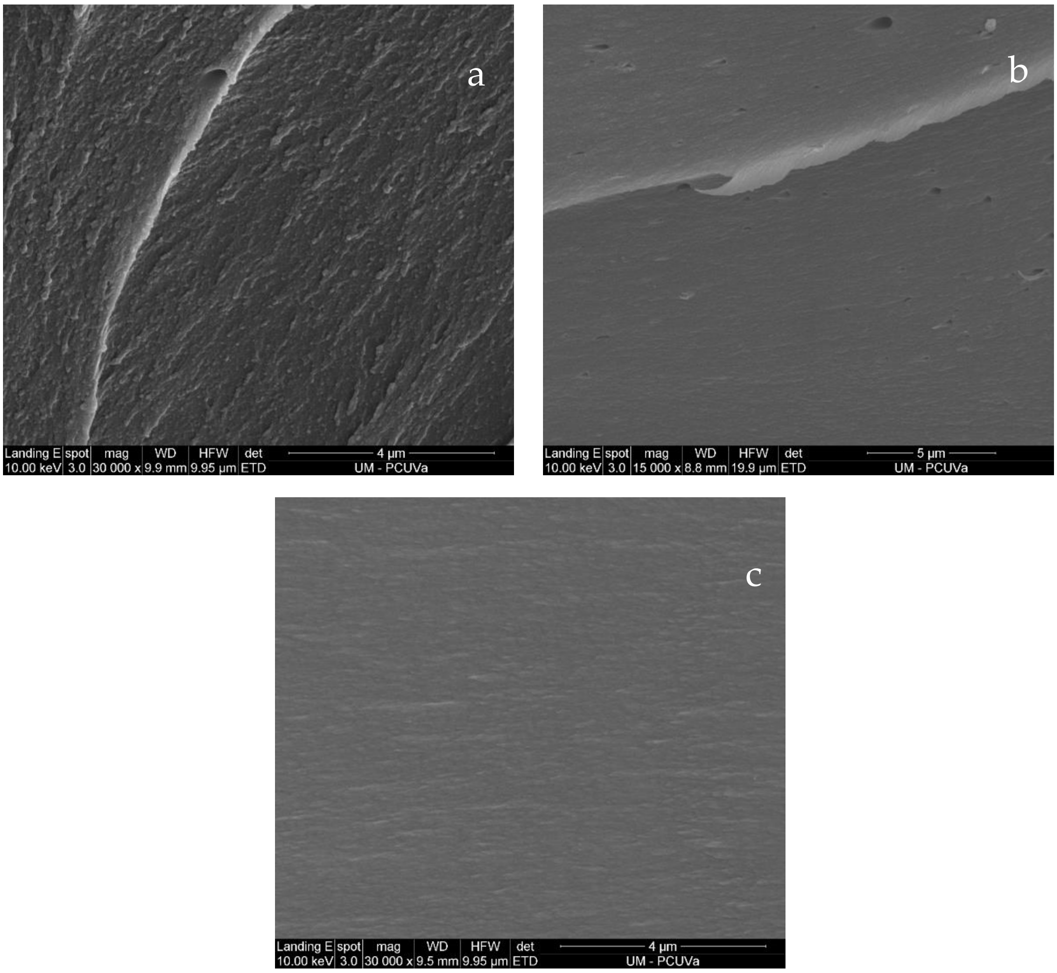

Figure S9. SEM micrographs of neat polyimide membranes: (**a**) Matrimid, (**b**) 6F6F, and (**c**) 6FTMPD.

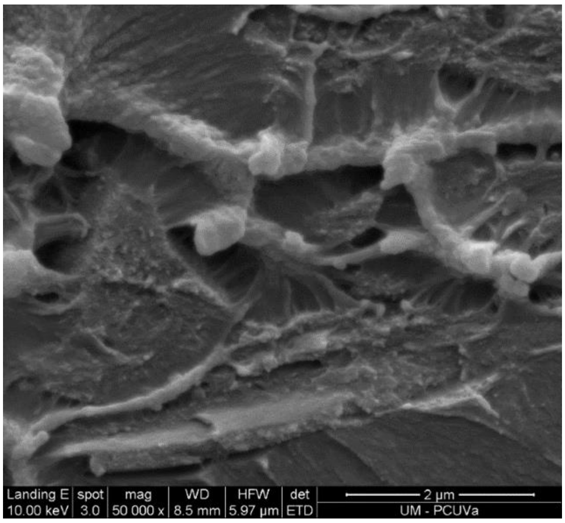

Figure S10. SEM micrograph of Matrimid-40.

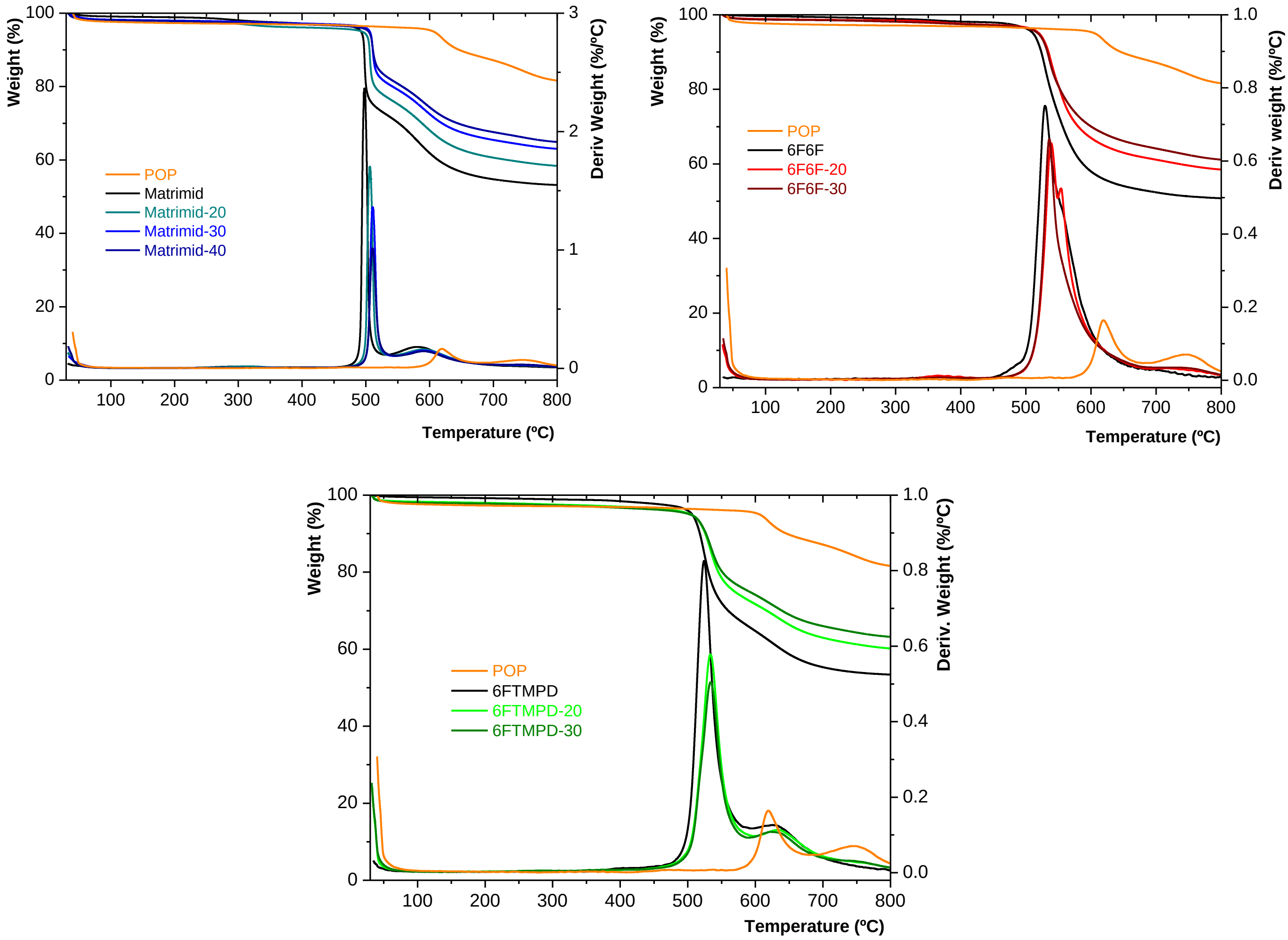

Figure S11. TGA curves of Matrimid (**top left**), 6F6F (**top right**) and 6FTMPD (**bottom**) of MMMs.

S5. Gas separation properties of MMMs

Table S1. Permeability (P, Barrer), diffusivity (D, $cm^2\ s^{-1} \times 10^8$) and solubility (S, $cm^3(STP)\ cm^{-3}\ atm^{-1}$) coefficients for 135TPB-DAFO POP-based MMMs and neat polyimide membranes at 30 °C and 3 bar feed pressure.

Membrane	Thickness (μm)		He	N_2	O_2	CH_4	CO_2	$\alpha O_2/N_2$	$\alpha CO_2/CH_4$	$\alpha CO_2/N_2$
**Matrimid**	54	P	22	0.25	1.60	0.22	8.60	6.4	39.0	25.02
		D	-	0.32	1.60	0.066	0.64	5.0	9.7	1.60
		S	-	0.59	0.76	2.53	10.2	1.2	4.1	17.3
**Matrimid-20**	53	P	34	0.85	4.41	0.70	20.9	5.19	29.8	24.5
		D	-	0.59	2.82	0.15	1.10	4.78	7.33	1.86
		S	-	1.09	1.19	3.44	14.4	1.09	4.19	13.2
**Matrimid-30**	67	P	49	1.58	8.55	1.58	37.9	5.41	24.0	24.0
		D	-	1.16	4.45	0.29	1.89	3.77	6.52	1.63
		S	-	1.03	1.46	4.13	15.2	1.44	3.68	14.7
**Matrimid-40**	58	P	74	3.29	16.3	2.83	87.2	4.95	30.8	26.5
		D	-	2.10	7.28	0.46	3.21	3.47	6.98	1.53
		S	-	1.19	1.71	4.65	20.7	1.44	4.45	17.4
**6F6F**	61	P	156	3.6	18	1.7	71	4.57	37.7	19.0
		D	-	2.6	9.2	0.381	3.9	4.10	7.15	1.85
		S	-	1.05	1.49	3.39	13.8	1.10	5.32	10.2
**6F6F-20**	51	P	175	10.0	36.1	6.57	161	3.61	24.5	16.1
		D	-	5.30	15.4	1.16	5.51	2.90	4.75	1.04
		S	-	1.44	1.78	4.32	22.2	1.24	5.14	15.4
**6F6F-30**	69	P	205	13.6	54.3	10.0	261	3.99	26.1	19.2
		D	-	8.34	21.6	2.33	13.6	2.59	5.84	1.63
		S	-	1.24	1.91	3.27	14.5	1.54	4.43	11.7
**6FTMPD**	48	P	328	25	94	19	444	3.82	22.0	17.7
		D	-	9.3	29	2.3	14	3.1	6.1	1.5
		S	-	2.04	2.46	6.28	24.1	1.2	3.8	12
**6FTMPD-20**	57	P	400	48.5	165	44.2	832	3.40	18.8	17.1
		D	-	18.3	58.1	5.0	22.5	3.17	4.5	1.23
		S	-	2.01	2.16	6.72	28.1	1.08	4.18	14.05
**6FTMPD-30**	70	P	557	81.1	280	84.6	1450	3.45	17.1	17.9
		D	-	25.4	74.2	7.4	39.4	2.92	5.32	1.55
		S	-	2.43	2.87	8.93	28.0	1.18	3.13	11.5

Table S2. Permeability (P, Barrer) and ideal selectivity for 135TPB-DAFO POP-based MMMs and neat polyimide membranes at 30 °C and 3 bar feed pressure.

Membrane	C_2H_6	C_2H_4	C_3H_8	C_3H_6	$\alpha C_2H_4/C_2H_6$	$\alpha C_3H_6/C_3H_8$	Reference
**6F6F** [a]	0.48	2.1	0.056	0.89	4.4	16	[2]
**6F6F** [b]			0.050	0.85		17	[3]
**6F6F**	0.345	1.52			4.4		This study
**6F6F-20**	2.59	9.55	0.219	4.0	3.7	18.3	This study
**6FTMPD** [a]	20 [c]	58 [c]	2.73	30	2.9	11	[4]
**6FTMPD**	16.0	48.6	1.69	26.2	3.04	15.5	This study
**6FTMPD-20**	26.9	77.3	5.53	58.1	2.87	10.5	This study

[a] 35 °C and 3.8 bar feed pressure, [b] 35 °C and 2 bar feed pressure, [c] 50 °C and 2 bar feed pressure

References.

1. Esteban, N.; Ferrer, M. L.; Ania, C. O.; de la Campa, J. G.; Lozano, Á. E.; Álvarez, C.; Miguel, J. A. Porous Organic Polymers Containing Active Metal Centers for Suzuki–Miyaura Heterocoupling Reactions. *ACS Appl. Mater. Interfaces* **2020**, *12*, 56974–56986, doi:10.1021/acsami.0c16184.
2. Staudt-Bickel, C.; Koros, W. J. Olefin/paraffin gas separations with 6FDA-based polyimide membranes. *J. Memb. Sci.* **2000**, *170*, 205–214, doi:10.1016/S0376-7388(99)00351-8.
3. Das, M.; Koros, W. J. Performance of 6FDA–6FpDA polyimide for propylene/propane separations. *J. Memb. Sci.* **2010**, *365*, 399–408, doi:10.1016/j.memsci.2010.09.029.
4. Zhang, C.; Dai, Y.; Johnson, J. R.; Karvan, O.; Koros, W. J. High performance ZIF-8 / 6FDA-DAM mixed matrix membrane for propylene / propane separations. *J. Memb. Sci.* **2012**, *389*, 34–42, doi:10.1016/j.memsci.2011.10.003.
